# Differential Cortical Activations Among Young Adults Who Fall Versus Those Who Recover Successfully Following an Unexpected Slip During Walking

**DOI:** 10.3390/brainsci15070765

**Published:** 2025-07-18

**Authors:** Rudri Purohit, Shuaijie Wang, Tanvi Bhatt

**Affiliations:** 1 Department of Physical Therapy, College of Applied Health Sciences, University of Illinois, Chicago, IL 60612, USA; rpuroh2@uic.edu (R.P.); sjwang4@uic.edu (S.W.); 2 Ph.D. Program in Rehabilitation Sciences and Neuroscience, Department of Physical Therapy, College of Applied Health Sciences, University of Illinois, Chicago, IL 60612, USA

**Keywords:** beta power, cerebral cortex, slip, falls, sensorimotor processing

## Abstract

**Background:** Biomechanical and neuromuscular differences between falls and recoveries have been well-studied; however, the cortical correlations remain unclear. Using mobile brain imaging via electroencephalography (EEG), we examined differences in sensorimotor beta frequencies between falls and recoveries during an unpredicted slip in walking. **Methods**: We recruited 22 young adults (15 female; 18–35 years) who experienced a slip (65 cm) during walking. Raw EEG signals were band-pass filtered, and independent component analysis was performed to remove non-neural sources, eventually three participants were excluded due to excessive artifacts. Peak beta power was extracted from three time-bins: 400 milliseconds pre-, 0–150 milliseconds post and 150–300 milliseconds post-perturbation from the midline (Cz) electrode. A 2 × 3 Analysis of Covariance assessed the interaction between time-bins and group on beta power, followed by Independent and Paired *t*-tests for between and within-group post hoc comparisons. **Results:** All participants (*n* = 19) experienced a balance loss, seven experienced a fall. There was a time × group interaction on beta power *(p* < 0.05). With no group differences pre-perturbation, participants who experienced a fall exhibited higher beta power during 0–150 milliseconds post-perturbation than those who recovered *(p* < 0.001). However, there were no group differences in beta power during 150–300 milliseconds post-perturbation. **Conclusions**: Young adults exhibiting a greater increase in beta power during the early post-perturbation period experienced a fall, suggesting a higher cortical error detection due to a larger mismatch in the expected and ongoing postural state and greater cortical dependence for sensorimotor processing. Our study results provide an overview of the possible cortical governance to modulate slip-fall/recovery outcomes.

## 1. Introduction

Falls among older adults are a global health concern [1] as they often result in health consequences like fractures, head injuries, and long-term disability [2,3,4,5]. Up to 40% of falls in older adults occur from environmental perturbations like slipping [6]. Although slip-related falls can be multifactorial, deficits in reactive balance control, i.e., the ability to restore balance following unpredicted perturbations, are postulated to be key contributing factors for high fall risk among older adults [7,8,9,10]. Reactive balance responses elicited by unpredicted perturbations include feet-in-place (hip or ankle strategies) [11] or change-in-support responses (compensatory stepping or grasping) [12] and are known to be affected by physiological aging and/or the presence of pathology [10,13,14,15,16,17]. Moreover, greater impairments in reactive balance control seen among older adults are postulated to be related to twofold higher fall risk than young adults [7,10,18,19,20,21]. Thus, understanding factors associated with impaired reactive balance responses could assist in developing effective strategies for fall prevention.

Mechanisms of failed reactive balance responses that result in a fall were commonly studied using biomechanical [22,23,24] and neuromuscular variables [25,26,27]. Specifically, studies identified that older adults who fall upon unpredictable slips during walking demonstrate worse reactive control of the center of the mass state (its position and velocity) and lower limb support at recovery limb touchdown than those who successfully recover balance without falling [22,24]. Such differences in biomechanical measures are postulated to arise from variations in the neuromuscular control of reactive balance. For instance, studies showed that older adults who fell upon unpredictable slips during walking demonstrated delayed onset of knee muscle activations, higher levels of agonist-antagonist coactivations in the slipping lower limb, and fewer interlimb muscle synergies than those who recovered successfully [25,26]. These differences in neuromuscular activation patterns observed between falls and recoveries are postulated to arise from differential modulations in cortical processes involved in reactive balance recovery [28,29].

Over the past decade, around 30 studies used mobile brain imaging techniques like electroencephalography (EEG) to examine cortical processes involved in reactive balance recovery [30,31,32,33,34,35]. These EEG studies commonly assessed two cortical outcomes during reactive balance tasks: perturbation-evoked potentials, mainly the balance potential: N1 (a negative deflection ~100–200 ms post-perturbation) [36,37,38] and cortical frequencies, mainly the beta frequency (oscillations occurring between 13 and 30 Hz) [30,31,33,39]. Results from EEG studies examining N1 during unpredicted perturbations in standing indicated increases in N1 amplitude with perceived postural threat (e.g., perturbation magnitude, ground height) [37,40,41]. Other EEG studies assessed changes in beta frequencies (13–30 Hz) and showed that sensorimotor beta frequency power increases with perturbation magnitude and is higher in adults with lower reactive balance (e.g., older adults) [31,42]. In summary, previous studies suggested that the sensorimotor cortex plays a role in processing perturbation characteristics and detecting errors in the central sensorimotor set to potentially modulate recovery responses. However, most previous studies examined stance perturbations and were restricted to young adults [30,31,42]. A couple of recent studies examined stance perturbations in older adults and showed that higher post-perturbation sensorimotor beta power was associated with worse reactive balance performance (e.g., longer movement latency or lower reactive stepping threshold) [39,42]. However, falls resulting from failed reactive balance responses might be more common during walking than during quiet standing, especially in ambulatory older adults [43]. Hence, understanding cortical mechanisms during walking might hold strong significance for populations at high risk of falling. To date, only two pilot studies have examined cortical frequencies during unpredicted walking perturbations using split-belt treadmills in young adults [32,44]. Although split-belt treadmills are portable and clinically adaptable, overground platforms mimic real-life slips, as outcomes are modulated by individual gait parameters. Moreover, all previous EEG studies delivered perturbations with relatively low perturbation intensities, possibly insufficient to elicit falls and, thus, could not differentiate the mechanistic correlates of falls versus successful recoveries [30,31,42]. To our knowledge, no previous study examined differences in cortical outcomes between falls and recoveries during overground walking perturbations such as slips or trips.

As a first step to understanding the potential role of the cerebral cortex in preventing falls, we examined changes in sensorimotor cortical beta power between falls and recoveries using the mobile brain imaging technique of EEG. Based on previous evidence suggesting that higher beta power is associated with worse reactive balance performance [31,33,45], we hypothesized that individuals who exhibited a higher pre-to-post increase in sensorimotor beta power would experience a fall upon an unpredicted slip during walking than those who successfully recovered balance without falling. Findings from the current study could help us understand whether there is a cortical basis for falling and if there is a need for future studies to explore cortical correlations of falls in people at high risk.

## 2. Materials and Methods

### 2.1. Participants

We recruited 22 healthy young adults (15 females, age: 18–35 years, weight: 70.27 ± 14.49 kg, and height: 1.67 ± 0.9 m) from the University of Illinois Chicago campus. Most participants (20 of 22) identified themselves as right-hand, right-foot dominant and had either normal or corrected vision (20/20 scoring on Snellen’s chart for visual acuity). Participants were excluded if they had any history or present musculoskeletal, cardiovascular, or neurological disorders or injuries, underwent surgeries in the past 3 months, or hospitalizations in the past 6 months. Participants were also excluded if they weighed over 115 kilograms, as this was the harness limit for the slipping experiment. Due to high standard deviations in the electrocortical signals, three participants were excluded from the analysis. Hence, a total of 19 participants were included. This study was approved by the Institutional Review Board at the University of Illinois, Chicago, and all participants provided written consent before enrollment.

### 2.2. Experimental Setup

The experimental setup consisted of a 7 m overground walkway embedded with a pair of low-friction, movable platforms mounted to supporting frames with linear ball bearings [15,46,47]. The supported frame of the sliding devices was bolted to a force plate (OR6-5, AMTI, Newton, MA, USA) to detect ground reaction forces [22,48]. The movable platforms were locked during walking trials and released during the slip trials using a computer-controlled mechanism, which unlocked the platform within 50 milliseconds of touchdown (Ground reaction force > 10 N) of the desired slipping limb [22,49]. Upon release, the platform was free to slide up to a preset distance of 65 cm [50,51].

### 2.3. Experimental Protocol

Participants donned a full-body safety harness to protect themselves from falling to the ground in the event of a slip. The safety harness was attached to a load cell for detecting falls, which was then mounted to an overhead trolley. Participants were instructed to walk at their self-selected comfortable pace throughout the experiment. This approach was adopted to emulate regular gait behavior and has been used in prior slip-perturbation studies to capture ecologically valid responses [22,52]. While speed was not explicitly controlled in this study, all participants walked under standardized instructions within a consistent laboratory setup. Although walking speed might influence neuromechanical and cortical outcomes, we accounted for this by including gait speed as a covariate in the EEG analyses. Participants first walked on the overground walkway for three trials (each trial = 10 seconds). Following baseline trials, participants were instructed about the possible occurrence of a slip under either limb in the upcoming trials without providing any warning about the exact timing or the nature of the slip. Participants were instructed to try their best to recover their balance and continue walking if they experienced a slip. After achieving the desired foot placement, a unilateral, forward slip was delivered under the right limb by releasing the right movable platform while walking.

### 2.4. Data Collection, Processing, and Analysis

Kinematic data were collected using an eight-camera motion capture system (Motion Analysis, Qualisys AB, Gothenburg, Sweden). A set of 30 reflective markers: 26 placed on bilateral bony landmarks, 2 on movable platforms, and 2 at fixed locations on the walkway, was used to record full-body kinematics [15,53]. The marker trajectories were low-pass filtered at specific frequencies (range 4.5–9 Hz) using a zero-lag fourth-order Butterworth filter [22]. The kinematic data were collected at 120 Hz, and the ground reaction force and load-cell data were collected at 600 Hz and synchronized with the kinematic data.

Brain activity was recorded using a set of 32-channel gel-based active recording electrodes with placement based on the international 10–20 system (g.tec neurotechnology, USA, Inc. Castleton-On-Hudson, NY, USA) [32]. Electrocortical signals from 32 channels were referenced to the earlobe sensor clip, and the ground electrode was located at the anterior fontanelle. A biosignal amplifier recorded the signals at 500 Hz with a 24-bit resolution and an in-built antialiasing filter set to 552 Hz. The signals from the ground electrode and all channels were recorded with an in-built impedance of less than 10 KΩ. A transistor-transistor-logic signal was delivered to the device to synchronize the EEG data with ground reaction force, load cell, and kinematic data. For this study, we focused our cortical beta power analysis on the Cz electrode, located over the midline sensorimotor cortex, based on prior studies demonstrating that Cz captured reliable beta oscillatory dynamics during balance perturbations and is frequently used in reactive balance literature [31,42,54,55]. These studies showed that beta activity at Cz is particularly sensitive to sensorimotor integration processes following destabilizing events.

Offline electrocortical signals were preprocessed and analyzed via EEGLAB version 2023.0 functions and custom MATLAB R2024b scripts. EEG signals were digitally filtered between 0.5 Hz and 100 Hz to remove baseline drift and high-frequency noise. Next, channels containing high-frequency noise (standard deviation > 4), flattened line (time > 5 s), and poorly correlated with other channels (r < 0.8) were automatically rejected using the EEGLAB function (pop_clean_rawdata). Independent component analysis (ICA) was conducted to correct the eye-blink, cardiac, muscle, and movement artifacts in the EEG signals (time > 200 s) for each subject. The corrected EEG signals were divided into single-trial epochs of 10 seconds and aligned with the motion and force data using the transistor-transistor-logic signal. For this study, we used single-trial epoch analysis for assessing changes in beta power between falls and recoveries. Although single-trial analysis is a relatively newer and less commonly used technique for the analysis of electrocortical signals [56,57,58], this technique can provide precise temporal information regarding event-related cortical differences between two conditions. Further, previous studies have shown that young adults can adapt and change to overground slips while walking in as little as one trial; thus, average-trial analysis could mask the perturbation-outcome-dependent cortical activations seen in the novel slip trial. Such adaptations can alter central set mechanisms and neuromuscular strategies in subsequent trials, thereby influencing cortical activity. While individuals may not retain conscious memory of the slip, neuromotor adaptations have been shown to persist even weeks later [48], limiting the generalizability of repeated trials for examining naïve cortical responses. Thus, we limited our analysis to the first slip exposure to isolate neural mechanisms during unadapted, reactive balance responses. Further, to reduce the influence of prior adaptation, we confirmed that no participants had experienced a fall or slip-related event in the past month and limited analysis to the first laboratory-induced slip to isolate unadapted cortical mechanisms.

We conducted a time-frequency analysis on the corrected electrocortical signal to assess changes in oscillatory power in the beta frequency range (13–30 Hz) before and after perturbation onset. The beta frequency range was specifically selected as the focus for this study based on previous literature suggesting the involvement of beta frequencies in sensorimotor information processing [30,33,34,35,59,60]. The time range from 1000 milliseconds before to 1000 milliseconds after slip perturbation onset was selected to extract beta oscillatory power in single-trial epochs using wavelet time-frequency analysis (EEGLAB function: pop_newtimef). A tapered Morlet wavelet was used to measure the power (amount of energy per unit of time). For each frequency in the beta range (13–30 Hz), power was quantified as the square of the amplitude of the Fast Fourier Transformation at that frequency. This enabled us to calculate the dynamic change in beta oscillations in a selected range of frequencies relative to the perturbation (slip) onset, known as the event-related spectral perturbation (ERSP). ERSP values were calculated for each trial at 200 time points 10 milliseconds apart, and 10 linearly spaced frequencies from 13 Hz to 30 Hz based on the spectral and temporal resolution. Following this, the single-trial ERSP values across four sampled frequencies within the beta range (centered on 15 Hz, 20 Hz, 24 Hz, and 28 Hz) were averaged to obtain a single waveform of beta power. We subtracted the mean baseline log spectrum (1000 to 800 milliseconds prior to the perturbation onset) from each spectral estimate, producing the baseline normalized time-frequency distribution. This process of data analysis was adopted from a recent article [31] that helped us remove filters from the data, including high-frequency artifacts (>30 Hz) and low-frequency artifacts (<13 Hz), without filtering the raw data before calculating the ERSPs.

### 2.5. Outcome Measures

#### 2.5.1. Perturbation Outcome

Each trial was classified as either a backward loss of balance with a fall or a recovery. A backward loss of balance occurred when the participant initiated a backward compensatory step in response to a forward perturbation and when the contralateral trailing limb landed posterior to the slipping limb at recovery touchdown. The outcome of a slip trial was classified as a fall if the peak load-cell force measured through the harness system exceeded 30% of the participant’s body weight upon slipping [61]. The fall threshold was selected based on previous work by Pai and colleagues, who validated this objective criterion for fall detection in both young and older adults using overground slip paradigms [22,61]. These studies demonstrated that the 30% threshold is highly sensitive and specific for distinguishing true fall events from recoveries and has been consistently employed in perturbation-based balance research. While this criterion has shown applicability across a broad range of body types and genders, it is important to note that the harness system imposes a safety-related upper weight limit of approximately 115 kg, which might limit generalizability to individuals weighing above 115 kg. We also used the video recording to cross-verify the perturbation outcome [62] and no inconsistencies were observed.

#### 2.5.2. Cortical Outcomes

Pre-perturbation beta power: Pre-perturbation beta power was quantified as the peak power across four sampled frequencies within the beta range (centered on 15, 20, 24, and 28 Hz) detected during 400 milliseconds before perturbation onset, focused over the Cz electrode [31].

Post-perturbation beta power: Post-perturbation beta power was quantified as the peak power across four sampled frequencies within the beta range (centered on 15, 20, 24, and 28 Hz) detected during two time-bins: 0–150 milliseconds and 150–300 milliseconds post perturbation onset focused over the Cz electrode [31]. These time-bins were chosen based on literature and corresponded with time events of reactive balance recovery processes, including recovery limb liftoff (~150 milliseconds after perturbation onset) and recovery limb touchdown (~300 milliseconds) [39,52,63,64].

#### 2.5.3. Biomechanical Outcomes

We assessed two key biomechanical variables: Center of mass (COM) state stability and vertical limb support post-perturbation, based on previous studies showing that both these variables can together predict >90% of slip-related falls [22,65].

Post-perturbation COM state stability: COM state stability was the shortest distance between the center of mass state (its position and velocity relative to the base of support) to the computational threshold against balance loss during slips [66]. Using the rear edge of the base of support (the heel of the slipping limb) as a reference, the center of mass state was used to calculate its instantaneous stability in two instances: post-slip liftoff (LO) of the recovery limb and its touchdown (TD). If the stability value was <0, the center of mass state was below the threshold, indicating a greater possibility of backward balance loss and higher fall risk.

Post-perturbation vertical limb support: Vertical limb support was quantified by the hip height, defined as the vertical distance between the midpoint of bilateral hips to the floor, which was determined from the anterior–superior iliac spine markers. Hip height (meters) was calculated at LO and TD of the recovery limb and normalized by each participant’s body height (meters) [16,67], thus resulting in a dimensionless variable. Higher values indicated greater vertical limb support.

### 2.6. Statistical Analysis

The Shapiro–Wilk normality test was used to check the distribution of pre- and post-perturbation beta power. The perturbation outcome, i.e., fall and recovery, was a dichotomous variable with 1 assigned for a fall and 0 assigned for a recovery. A 2 × 3 mixed model Analysis of Covariance was used to test the interaction of group (fall and recovery) and time-bin (pre-, 0–150 milliseconds post, and 150–300 milliseconds post-perturbation) and their interaction on beta power with gait speed as a covariate. Post hoc comparisons were performed using Paired and Independent *t*-tests for within and between-group pairwise comparisons. Independent *t*-tests were used to compare the differences in biomechanical outcomes (COM state stability and vertical limb support) between the two groups. All analyses were conducted in Statistical Package for the Social Sciences (SPSS) software, version 27, with an alpha value set at 0.05.

## 3. Results

**Figure 1:**
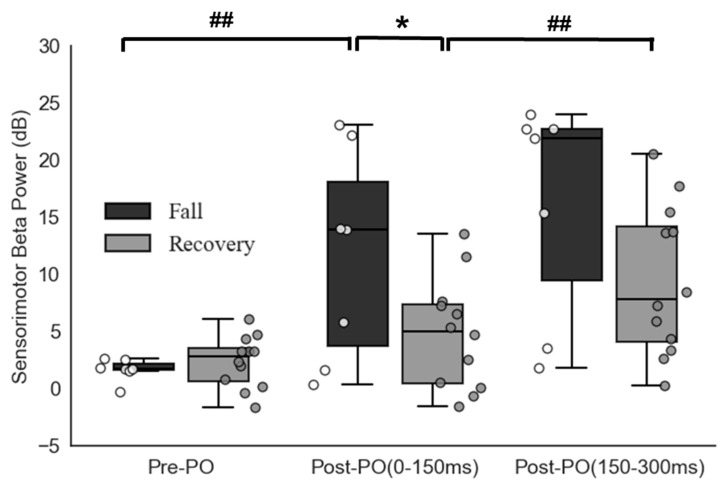
Box plots and individual data points representing the distribution of peak beta power focused at the Cz electrode between two groups: falls (*n* = 7) and successful recoveries (*n* = 12) and three time points: pre-perturbation onset (Pre-PO), i.e., 400 milliseconds before perturbation onset and early post-perturbation onset (post-PO), i.e., 0–150 milliseconds after and late post-perturbation onset (post-PO), i.e., 150–300 milliseconds after. Between groups, significant differences are represented as * for *p* < 0.05; significant differences at different time points within-groups are represented as ## for *p* < 0.01. Abbreviations: PO—Perturbation onset, dB—Decibel (units for beta power).

**Figure 2:**
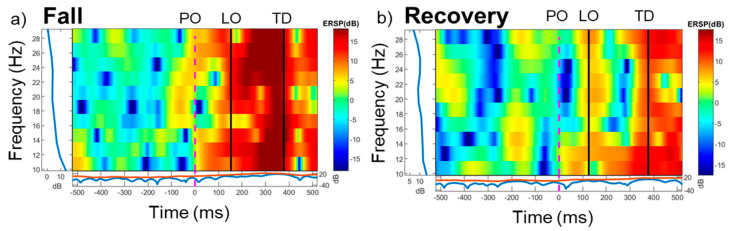
Event-related spectral perturbations (ERSPs) focused at the Cz electrode in the beta frequency range (13–30 Hz) for two representative participants who experienced: (**a**) A Fall and (**b**) A Recovery following an unpredicted slip during walking. (**a**) shows a sharp increase in power (dB) throughout the beta frequency range (13–30 Hz), whereas (**b**) shows a modest increase in power (dB) throughout the beta frequency range. Notably, pre-perturbation beta power is visible similarly across the beta frequency range. Abbreviations: PO (Perturbation onset), LO (Lift-off) of the recovery limb, and TD (Touchdown) of the recovery limb.

Out of 19 included, 7 participants experienced a fall upon slipping, whereas 12 recovered. The Shapiro–Wilk normality test showed the normality assumption was met for all outcomes (*p* > 0.05). All participants experienced a backward loss of balance upon novel slip exposure. The 2 × 3 ANCOVA showed a group × time interaction on beta power [F (2, 46) = 178.21, *p* < 0.001). There were no differences in pre-perturbation beta power between the two groups [Falls: pre-perturbation = 3.65 ± 1.30 dB, Recoveries = 3.78 ± 1.15 dB (t (15) = 0.24, *p* = 0.81)] (Figure 1). Within the fall group, increased beta power was observed during the 0–150 milliseconds post-perturbation compared to pre-perturbation [Falls: t (6) = −2.83, *p* < 0.01], whereas no significant changes were seen within the recovery group [ t (11) = −1.596, *p* > 0.05]. Between the two groups, the fall group showed higher beta power during 0–150 milliseconds post-perturbation compared to those who recovered successfully [t (11) = 2.116, *p* < 0.05]. Between the two time-bins, the recovery group showed an increase in beta power from 0–150 to 150–300 milliseconds [t (11) = −3.720, *p* = 0.002], whereas there were no differences seen within the fall group [t (6) = −1.932, *p* > 0.05. Representative ERSPs from one participant who fell and one who recovered can be seen in Figure 2.

For the biomechanical outcomes, falls showed lower post-perturbation COM state stability at recovery limb liftoff and touchdown than recoveries (*p* < 0.05) (Table 1). Further, falls exhibited a trend toward lower limb support at liftoff than recoveries, though this difference did not reach statistical significance (*p* = 0.06) (Table 1). Lastly, we observed a moderate negative correlation between post-perturbation beta power and COM stability at recovery limb liftoff and touchdown [r = −0.57, *p* = 0.021 (LO); r = −0.56, *p* = 0.025 (TD)].

## 4. Discussion

This study examined differences in sensorimotor cortical beta power between falls and recoveries following an unpredictable slip while walking. Our results were in partial alignment with our hypothesis and showed that young adults who exhibited a greater increase in beta power pre- to post-perturbation (0–150 milliseconds) experienced a fall post-slipping, with no group differences in beta power pre-perturbation and 150–300 milliseconds post-perturbation. The group differences in sensorimotor cortical beta power at 0–150 milliseconds post-perturbation seen in this study could not be explained by the initial motion state (i.e., pre-perturbation cortical or biomechanical variables). In turn, the observed post-perturbation cortical differences could be explained by group differences in post-perturbation biomechanical variables, which are postulated to arise from differences in neuromuscular responses (muscle onset latencies or muscle synergies) between falls and recoveries [25,26].

*Pre-perturbation cortical activity might not influence the perturbation outcome (fall vs. recovery).*

No significant differences were found in pre-perturbation beta power between young adults who fell and those who recovered upon slipping, suggesting that pre-perturbation cortical activity might not influence the post-perturbation outcome for a novel, unpredicted perturbation. This postulation can be explained based on two themes of evidence. First, like our findings, previous studies in young adults have shown no pre-perturbation differences in beta power, with significant differences post-perturbation seen with variable perturbation magnitudes and variable balance abilities [30,31,42]. Thus, our findings suggest that pre-perturbation beta dynamics might not influence the post-perturbation outcome. Second, studies have suggested that the risk of falling upon an external perturbation can be reduced through proactive adjustments to prepare the sensorimotor system for anticipated stimulus, as well as reactive adjustments to the ‘central set’ (internal model) [68,69]. These adjustments enable neuromuscular changes to modify the body’s center of mass (COM) state for balance recovery [69,70,71,72]. Proactive and reactive adjustments in this central set are postulated to arise from or be modified by various factors (e.g., cognitive state, initial sensorimotor conditions, or warning of a perturbation) [68,69]. In this study, participants were warned about the possibility of an upcoming perturbation; however, the timing and context of the perturbation were unknown. Given this unpredictability, the cerebral cortex might be less likely to induce adjustments in the central set before the perturbation onset and, thus, might not govern the following perturbation outcome (fall vs. recovery).

*A significant increase in sensorimotor beta power during the early post-perturbation period was seen in individuals who fell.*

Fallers exhibited increased sensorimotor beta power following perturbation onset compared to those who recovered successfully. Previous studies have postulated that upon the onset of an unpredicted perturbation, afferent inputs from the somatosensory, visual, and vestibular receptors are relayed to the primary somatosensory cortex for processing of perturbation characteristics, sensory reweighting, and multisensory integration [73,74,75,76]. A crucial aspect of sensorimotor processing is error detection, i.e., detection of a mismatch between the expected postural state based on the central set (internal model) and the ongoing postural state based on sensorimotor information relayed in real-time [77,78,79,80,81]. It is postulated that several factors can result in large errors in the detection of the expected and ongoing postural state. These factors include age or pathology-related alterations in sensorimotor reweighting and integration, [82,83] structural and functional neurological status [82,84], cognitive load or attentional demands, [85] and task complexity and environmental factors [72]. Along these lines, the increase in beta power during the early post-perturbation period seen in young adults in this study could stem from alterations in cognitive load or attentional demands, in addition to task complexity and environmental factors. Specifically, it can be postulated that a group of young adults might have perceived the task as highly complex, inducing a high postural threat, or may have experienced alterations in cognitive load. Increased beta power during the early post-perturbation period seen in fallers could also reflect heightened cortical responses to perceived postural threat. Prior studies have shown that psychological states such as fear of falling or anticipation of instability can amplify cortical beta and N1 responses, particularly in balance-threatening tasks [40,41]. Although participants in the current study were informed about a potential slip without knowing its exact timing, their individual perception of the threat may have varied and influenced cortical activity, especially during the early processing window. This variability, while inherent to ecological paradigms, could contribute to differences in cortical engagement during slips.

*Potential role of beta frequency oscillations in the context of reactive balance responses and falls.*

During the late post-perturbation period, there was a significant increase in beta power seen among those who recovered successfully, whereas no differences were seen among those who fell, which could be explained based on the role of beta frequency in the motor control domain. Specifically, the increase in beta power in the recovery group could indicate the CNS’s ability to command timely motor responses for reactive balance recovery (e.g., compensatory stepping). Previous studies have shown that beta oscillations are strongly associated with motor preparation and motor command generation, especially in the sensorimotor cortex [86,87,88,89]. Beta power is also linked to motor preparation, with increased activity often reflecting a readiness to engage motor control mechanisms for postural adjustments [59,79,88,90]. In this study, the recovery group likely exhibited more efficient motor control strategies, allowing them to generate appropriate corrective movements quickly, which are crucial processes involved in successful postural recovery [91]. Additionally, increased beta power during the recovery period seen in this study is consistent with previous study findings suggesting that beta oscillations reflect the brain’s engagement in controlling and coordinating motor responses for postural stability after perturbations [31,59,90]. In contrast, the fall group might have failed to generate such motor commands in a timely and efficient manner, leading to failed reactive balance recovery responses, as indicated by the lack of changes in beta power.

Correspondingly, the cortical changes during post-perturbation time periods in sensorimotor beta power between falls and recoveries could explain the biomechanical group differences seen in this study, i.e., lower COM stability seen at recovery limb touchdown among the fall group compared to the recovery group. The lower COM state stability at recovery limb touchdown seen in falls might arise from central cortical errors in modulating slipping limb control and/or recovery limb control [92,93,94]. This postulation can be supported by two pieces of evidence. First, poor slipping limb control is known to be a factor that can reduce vertical limb support, which is a known predictor of slip-related falls [65,95]. Second, central cortical errors in modulating the recovery limb control could explain the lower COM stability at recovery touchdown seen in fallers than in those who recovered [22]. Hence, it can be postulated that central cortical errors in modulating the recovery limb control might influence the succeeding perturbation outcomes, i.e., falls vs. recoveries.

While our study provides novel insights into the cortical mechanisms underlying reactive balance responses in healthy young adults, caution is warranted in generalizing these findings to older adults or individuals with neurological impairments. Age-related changes in sensorimotor integration, motor planning, and cortical efficiency can significantly alter cortical activation patterns during balance tasks [14,82]. For instance, older adults often exhibit delayed cortical responses and greater reliance on cortical control due to diminished peripheral feedback and slower neuromuscular activation [71,96,97,98,99]. Similarly, individuals with stroke or neurodegenerative conditions may demonstrate distinct cortical reorganization and compensatory mechanisms that influence both the timing and magnitude of beta oscillations [100,101,102,103,104]. As such, while our results offer foundational evidence for cortical modulation during slip-induced perturbations and lay the groundwork for comparison with fall-prone populations, future studies in clinical populations are essential to assess the translational relevance of these findings and to tailor interventions for fall prevention in high-risk groups.

### Study Limitations

Our results must be interpreted considering their limitations. First, we used single-trial analysis to assess differences in cortical activations between falls and recoveries between individuals as previous studies have shown that motor adaptation to overground slips can occur in as little as one trial [48,49,65]. Second, the current study only examined differences in sensorimotor beta power to unpredicted, novel slips. Hence, the results cannot be generalized to other perturbation types (e.g., trips, mediolateral perturbations). Further, we did not examine differences in neuromuscular activations, as it is well-established that older adults at high risk of falling (i.e., those who fell in the laboratory following a novel gait slip) demonstrate distinct neuromuscular characteristics (e.g., co-activations of agonists and antagonists) compared to those with low risk (i.e., who recovered following novel gait-slip) [25,26]. Although Cz is commonly used in balance and mobility EEG research due to its alignment with the central sensorimotor cortex [31,42,54,55], this approach might overlook potentially relevant activity in lateral motor areas such as C3 and C4. Future studies could benefit from a multichannel analysis or data-driven spatial filtering approaches to further explore the spatial characteristics of perturbation-related beta dynamics. Another limitation of this study is the lack of formal measurement of subjective threat or task-related anxiety, which has been shown to modulate cortical responses during balance tasks [40,41]. While participants were uniformly briefed about the possibility of slipping, their individual threat perception was not quantified. Future studies may benefit from integrating psychometric measures such as the State-Trait Anxiety Inventory or visual analog scales to better understand the interaction between cognitive-affective factors and cortical dynamics during reactive balance responses. Finally, we acknowledge that our study findings are based on a relatively small sample size (*n* = 19), which might limit statistical power and increase the risk of Type I or Type II errors. Although the sample was sufficient to detect significant differences in beta power between falls and recoveries, caution must be exercised when generalizing these results. The limited sample size may also reduce the robustness of observed associations and restrict the ability to perform subgroup analyses (e.g., based on sex or dominant limb). As such, these findings should be considered preliminary and hypothesis-generating. Future studies with larger, more diverse cohorts could validate these cortical activation patterns and further elucidate their relevance to real-world fall risk and rehabilitation strategies.

## 5. Conclusions

This study identified the differences in sensorimotor beta power between falls and recoveries following an unpredicted slip during walking, with results suggesting differential event-related cortical modulations based on reactive balance recovery processes. Young adults who showed a greater increase in beta power in the early post-perturbation period experienced a fall, suggesting a higher cortical error detection due to a larger mismatch in expected and ongoing postural state, along with greater dependence on cortical processes for sensorimotor processing. Young adults who showed a greater increase in beta power in the late post-perturbation period successfully recovered without falling, suggesting the influence of cortical commands for the execution of timely and effective balance recovery responses. The study findings suggest possible cortical governance to modulate perturbation outcomes following unpredicted slip perturbations. The study findings provide preliminary evidence on the potential role of the cerebral cortex in preventing falls and lay the foundational groundwork for future comparison with pathological populations.

## Figures and Tables

**Table 1:** Means and standard deviations of biomechanical outcomes between falls and recoveries.

Variables	Falls (*n* = 7) Mean (SD)	Recoveries (*n* =12) Mean (SD)	t	*p*-Value
**COM stability (LO)**	**−0.31 (0.09)**	**−0.17(0.10)**	**−3.69**	0.002 *
**COM stability (TD)**	**−0.55 (0.20)**	**−0.37 (0.14)**	**−2.41**	0.029 *
Hip height (LO)	0.52 (0.03)	0.50 (0.01)	1.66	0.06
Hip height (TD)	0.51 (0.03)	0.49 (0.01)	1.22	0.24

Abbreviations: COM: Center of mass; LO: Liftoff; TD: Touchdown; * = significant differences. The outcomes with significant differences are bolded. Both COM stability and hip height are dimensionless variables.

## Data Availability

The data presented in this study are available on request from the corresponding author due to ethical reasons.
